# Primary Immunodeficiencies in Russia: Data From the National Registry

**DOI:** 10.3389/fimmu.2020.01491

**Published:** 2020-08-06

**Authors:** Anna A. Mukhina, Natalya B. Kuzmenko, Yulia A. Rodina, Irina V. Kondratenko, Andrei A. Bologov, Tatiana V. Latysheva, Andrei P. Prodeus, Alexander N. Pampura, Dmitrii N. Balashov, Natalya I. Ilyina, Elena A. Latysheva, Ekaterina A. Deordieva, Oksana A. Shvets, Elena V. Deripapa, Irina N. Abramova, Olga E. Pashenko, Svetlana S. Vahlyarskaya, Natalya V. Zinovyeva, Sergei B. Zimin, Elena V. Skorobogatova, Elena B. Machneva, Daria S. Fomina, Maria G. Ipatova, Ludmila Yu. Barycheva, Ludmila S. Khachirova, Irina A. Tuzankina, Michail A. Bolkov, Natalya V. Shakhova, Elena M. Kamaltynova, Farida I. Sibgatullina, Marina N. Guseva, Raisa N. Kuznetsova, Anzhelika M. Milichkina, Areg A. Totolian, Natalia M. Kalinina, Evgenia A. Goltsman, Ekatherina I. Sulima, Anastasia Yu. Kutlyanceva, Anna A. Moiseeva, Anna L. Khoreva, Zoya Nesterenko, Elena V. Tymofeeva, A. Ermakova, Dilyara D. Proligina, Linara R. Kalmetieva, Gulshat A. Davletbaieva, Irina A. Mirsayapova, Olga A. Richkova, Ksenia P. Kuzmicheva, Maria A. Grakhova, Natalya B. Yudina, Ekaterina A. Orlova, Olga S. Selezneva, Svetlana G. Piskunova, Tatiana V. Samofalova, Tatiana V. Bukina, Anna D. Pechkurova, N. Migacheva, A. Zhestkov, Elena V. Barmina, Natalya A. Parfenova, Svetlana N. Isakova, Elena V. Averina, Irina V. Sazonova, Svetlana Yu. Starikova, Tatiana V. Shilova, Tatiana V. Asekretova, Roman N. Suprun, Elena I. Kleshchenko, Vladimir V. Lebedev, Elena V. Demikhova, Valerii G. Demikhov, Veronica A. Kalinkina, Alla V. Gorenkova, Svetlana N. Duryagina, Tatiana B. Pavlova, Vera M. Shinkareva, Irina V. Smoleva, Tatiana P. Aleksandrova, Zema V. Bambaeva, Marina A. Philippova, Elena M. Gracheva, Galina I. Tcyvkina, Alexey V. Efremenkov, D. Mashkovskaya, Irina V. Yarovaya, Valentina A. Alekseenko, Ivan V. Fisyun, Galina V. Molokova, Ekatherina V. Troitskya, Ludmila I. Piatkina, Elena V. Vlasova, O. Ukhanova, Ekaterina G. Chernishova, M. Vasilieva, Olga M. Laba, E. Volodina, Ekaterina V. Safonova, Kirill A. Voronin, Maria V. Gurkina, Alexander G. Rumyantsev, Galina A. Novichkova, Anna Yu. Shcherbina

**Affiliations:** ^1^Dmitry Rogachev National Research Center of Pediatric Hematology, Oncology and Immunology, Moscow, Russia; ^2^Russian Children's Clinical Hospital of the N.I. Pirogov Russian National Research Medical University, Ministry of Health of Russia, Moscow, Russia; ^3^National Research Center Institute of Immunology, Federal Biomedical Agency of Russia, Moscow, Russia; ^4^Speransky Children's Municipal Clinical Hospital #9, Moscow, Russia; ^5^Research and Clinical Institute for Pediatrics named After Academician Yuri Veltischev of the Pirogov Russian National Research Medical University of the Russian Ministry of Health, Moscow, Russia; ^6^Allergy and Immunology Centre, Clinical Hospital, Moscow, Russia; ^7^Sechenov First Moscow State Medical University, Moscow, Russia; ^8^Filatov Children's Municipal Clinical Hospital, Moscow, Russia; ^9^Stavropol State Medical University, Stavropol, Russia; ^10^Regional Pediatric Clinical Hospital, Stavropol, Russia; ^11^Institute of Immunology and Physiology—Ural Branch of the Russian Academy of Sciences, Ekaterinburg, Russia; ^12^Altai State Medical University, Barnaul, Russia; ^13^Department of Health of Tomsk Region, Tomsk, Russia; ^14^Regional Children's Hospital, Tomsk, Russia; ^15^Siberian State Medical University, Tomsk, Russia; ^16^Tatarstan Pediatric Republican Clinical Hospital, Kazan, Russia; ^17^Saint-Petersburg Pasteur Institute, Saint-Petersburg, Russia; ^18^Saint-Petersburg State Pediatric Medical University, Saint-Petersburg, Russia; ^19^Pediatric Polyclinic Department of Murmansk, Murmansk, Russia; ^20^Regional Pediatric Clinical Hospital, Nizhny Novgorod, Russia; ^21^Republican Children's Clinical Hospital, Republic of Bashkortostan, Ufa, Russia; ^22^Tyumen State Medical University, Tyumen, Russia; ^23^Regional Clinical Hospital No.1, Tyumen, Russia; ^24^Voronezh Regional Pediatric Hospital #1, Voronezh, Russia; ^25^Rostov-na-Donu Regional Pediatric Clinical Hospital, Rostov-na-Donu, Russia; ^26^Pediatric Polyclinic #2, Allergy and Immunology, Volgograd, Russia; ^27^Clinical Hospital “Mother and Child”, Samara, Russia; ^28^Samara State Medical University, Samara, Russia; ^29^Tver Regional Pediatric Hospital, Tver, Russia; ^30^Department of Health of Vladimir Region, Vladimir, Russia; ^31^Federal State Budgetary Scientific Research Institute of Fundamental and Clinical Immunology, Novosibirsk, Russia; ^32^Pediatric Polyclinic #2, Sevastopol, Russia; ^33^Pediatric Clinical Hospital #2, Omsk, Russia; ^34^Federal State Budgetary Educational Institution of Higher Education “South-Ural State Medical University” of the Ministry of Healthcare of the Russian Federation, Chelyabinsk, Russia; ^35^Pediatric Regional Clinical Hospital, Krasnodar, Russia; ^36^Ryazan State Medical University, Ryazan, Russia; ^37^Department of Health of Khanty-Mansi Autonomous Region—Yugra, Khanty-Mansi, Russia; ^38^Northern State Medical University, Arkhangelsk, Russia; ^39^Irkutsk Regional Pediatric Hospital, Allergy and Immunology, Irkutsk, Russia; ^40^Regional Pediatric Clinical Hospital, Belgorod, Russia; ^41^Regional Pediatric Clinical Hospital, Bryansk, Russia; ^42^Children's Republican Clinical Hospital of Buryatiya, Ulan-Ude, Russia; ^43^Regional Pediatric Clinical Hospital, Vologda, Russia; ^44^Department of Health of Vologda Region, Vologda, Russia; ^45^Regional Clinical Allergy and Immunology Center, Vladivostok, Russia; ^46^Pediatric Republican Clinical Hospital, Crimea, Russia; ^47^Lipetck Regional Clinical Hospital, Lipetck, Russia; ^48^Pediatric Polyclinic #12, Omsk, Russia; ^49^Krugloi Clinical and Research Center, Orel, Russia; ^50^Pediatric Clinical Hospital #13, Perm, Russia; ^51^Regional Pediatric Hospital, Perm, Russia; ^52^Pediatric Medical Center #1, Saratov, Russia; ^53^Regional Clinical Hospital #1, Ekaterinburg, Russia; ^54^Regional Clinical Hospital, Stavropol, Russia; ^55^Regional Pediatric Hospital, Tula, Russia; ^56^Postgraduate Institute for Public Health Workers, Khabarovsk, Russia; ^57^Center of Allergy and Clinical Immunology, Regional Clinical Hospital named after Professor S.I. Sergeev, Khabarovsk, Russia; ^58^Regional Pediatric Hospital, Yaroslavl, Russia; ^59^Clinical Center, Bryansk, Russia; ^60^Regional Clinical Center of Maternity and Childhood Protection, Krasnoyarsk, Russia

**Keywords:** primary immunodeficiency, epidemiology, genetics, PID registry, HSCT, IVIG

## Abstract

**Introduction:** Primary immunodeficiencies (PID) are a group of rare genetic disorders with a multitude of clinical symptoms. Characterization of epidemiological and clinical data via national registries has proven to be a valuable tool of studying these diseases.

**Materials and Methods:** The Russian PID registry was set up in 2017, by the National Association of Experts in PID (NAEPID). It is a secure, internet-based database that includes detailed clinical, laboratory, and therapeutic data on PID patients of all ages.

**Results:** The registry contained information on 2,728 patients (60% males, 40% females), from all Federal Districts of the Russian Federation. 1,851/2,728 (68%) were alive, 1,426/1,851 (77%) were children and 425/1,851 (23%) were adults. PID was diagnosed before the age of 18 in 2,192 patients (88%). Antibody defects (699; 26%) and syndromic PID (591; 22%) were the most common groups of PID. The minimum overall PID prevalence in the Russian population was 1.3:100,000 people; the estimated PID birth rate is 5.7 per 100,000 live births. The number of newly diagnosed patients per year increased dramatically, reaching the maximum of 331 patients in 2018. The overall mortality rate was 9.8%. Genetic testing has been performed in 1,740 patients and genetic defects were identified in 1,344 of them (77.2%). The median diagnostic delay was 2 years; this varied from 4 months to 11 years, depending on the PID category. The shortest time to diagnosis was noted in the combined PIDs—in WAS, DGS, and CGD. The longest delay was observed in AT, NBS, and in the most prevalent adult PID: HAE and CVID. Of the patients, 1,622 had symptomatic treatment information: 843 (52%) received IG treatment, mainly IVIG (96%), and 414 (25%) patients were treated with biological drugs. HSCT has been performed in 342/2,728 (16%) patients, of whom 67% are currently alive, 17% deceased, and 16% lost to follow-up. Three patients underwent gene therapy for WAS; all are currently alive.

**Conclusions:** Here, we describe our first analysis of the epidemiological features of PID in Russia, allowing us to highlight the main challenges around PID diagnosis and treatment.

## Introduction

Primary immunodeficiencies (PID)—also referred to as “inborn errors of immunity”—are rare disorders characterized by susceptibility to infection and a preponderance of autoimmunity, allergy, autoinflammation, and malignancies. According to the latest update of the International Union of Immunological Societies Experts Committee (IUIS) (1) classification, germline mutations in 430 genes cause 404 distinct phenotypes of immunological diseases, divided into 10 groups according to the type of immunological defect. Wide introduction of the molecular genetic techniques, including next-generation sequencing (NGS) (2), has led to the description of novel PID genes. This allows for a more precise assessment of clinical prognosis and for the choice of targeted therapy—or even gene therapy—as well as for family counseling (3).

Generally, PID are described as rare diseases. Yet their reported prevalence varies greatly in different countries, depending on many factors: from data collection methodology to objective epidemiological features. In European countries, the estimated prevalence of PID ranges from 2.7/100,000 in Germany, to 4.16-5.9/100,000 in Switzerland and the United Kingdom (UK), to 8/100,000 in France (3–7). These numbers are in the range of the "orphan diseases" category. Yet recent findings, in patients with mendelian susceptibility to mycobacterial diseases (MSMD) (8), suggest that the actual prevalence is much higher.

National PID registries (2, 3, 5), along with registries combining data for geographical regions (9, 10), have proven to be an important tool for assessing the clinical and epidemiological features of PID—as well as an instrument for facilitating PID collaboration and research, both within and between countries.

Several PID cohort study reports from Russia (11, 12) have been published recently, yet little has been known about the overall epidemiological features of PID in the heterogeneous Russian population. The aim of this study is to describe PID epidemiology in Russia, using a national registry.

## Materials and Methods

### Registry Structure

The Russian PID registry was established in 2017, as an initiative of the National Association of Experts in PID (NAEPID)—a non-profit organization facilitating collaboration amongst leading specialists in the field of primary immunodeficiencies in Russia. The registry is a secure on-line database, developed, and designed with the aim of collecting epidemiological, clinical, and genetic data of PID in Russia. It includes demographic data, clinical and laboratory details, molecular diagnosis, and treatment aspects of PID patients of all ages. Regular information updates allow for the collection of prospective data. The data is entered via an online registry form only; no paper-based documentation is needed. A group of trained managers at federal centers and doctors at regional hospitals enter the data in the database.

This article analyzes the data input into the registry from its inception until February 1, 2020.

At the time of the data analysis, PID variants were grouped according to the IUIS 2015–2017 classification (1) and did not include the newly added category of bone marrow failure (13). The database structure includes the following obligatory fields: demographic data, family history, diagnosis, genetic testing results, and ages of disease onset and diagnosis. The extended universal fields—including detailed clinical description and treatment data—are not mandatory at the time of the first registry of a patient, but are eventually requested. New entries are reviewed automatically, and no duplicate entries can be created. Human-factor errors are prevented by built-in quality assurance measures.

Patients can only be registered if the documenting center is part of the registry's collaborative team. Written informed consent is given by all registered patients or their legal guardians. Regularly updated reports on PID epidemiological data are published on the NAEPID Registry website http://naepid-reg.ru.

#### Registry Platform

The software platform used in the study was developed by Rosmed.info, using the PHP programming language. For database management, the Maria DB relational system (offshoot of the MySQL system) was utilized. Server Version: the 10.1.40-Maria DB Server and replication mechanism were used for back-up and improved performance; the server's contour and physical protection were compliant with Russian law regarding personal information protection.

### Centers

Russia is divided into 85 regions, which are grouped geographically into eight federal districts. Data on the PID patients residing in 83 of the federal regions has been accumulated in the registry, with the input of regional and tertiary centers. No patients residing in the other two regions (Chukotka and Tuva) were registered in the database. At the time of analysis, 69 regional medical centers and 5 university clinics—located in all 8 federal districts—have contributed to the collaborative work. Three tertiary immunology centers located in Moscow serve as the main reference centers. The diagnosis of the majority of the patients (2,488/2,728, 91%) has been confirmed in at least one of the tertiary centers.

### Patients

PID diagnosis was made according to the ESID diagnostic criteria (13). Patients with secondary immune defects were excluded. Although the registry collects data on all PID, 233 patients with selective IgA deficiency, and 106 patients with PFAPA (periodic fever, aphthous stomatitis, pharyngitis, adenitis) were not included in the current analysis.

The entire cohort of patients (2,728) was included in the epidemiological analysis—while, for the treatment description, we used only the updated information available for the 1,851 alive patients.

Genetic testing has been performed using the main molecular techniques, including Sanger sequencing, targeted next-generation sequencing (NGS), whole-exome and whole-genome sequencing, fluorescent *in situ* hybridization (FISH), multiplex ligation-dependent probe amplification (MLPA), and chromosomal microarray analysis (CMA), according to standard protocols.

### Data Verification

All data entered into the registry undergoes automatic verification for typing errors and is regularly checked by the database monitor for consistency and completeness.

### Terminology and Definitions

The actual age distribution was calculated only for the patients with updated information; the age of each patient was determined as the difference between their date of birth and the date of the last update.

Patients without any contact within the last 2 years were marked as “lost to follow-up.”

The diagnostic delay was estimated for all registered patients, in the nine most common PID categories, as the difference between the date of disease onset and the date of clinical diagnosis of PID.

Prevalence was estimated as the number of all registered PID cases, divided by the population of Russia or of each federal district; information was obtained from open resources^1^.

Incidence was estimated as the number of new PID cases diagnosed during each year, divided by the number of live births during that year in Russia; information was obtained from open resources.

Prevalence and incidence were expressed as the number of cases per 100,000 people.

Mortality rate, expressed in percentage, was estimated as the number of deceased patients divided by the number of all updated PID cases; lost-to-follow-up patients were excluded.

The category of “fully recovered” was not available at the time of analysis.

Patients from birth to 17 years, 11 months, and 29 days were counted as children. The rest were considered adults.

### Statistical Analysis

Demographic and epidemiological characteristics were described as average for the categorical variables, and median and range for the quantitative variables. To compare the prevalence of the diseases, the chi-squared test was used and a *p*-value of <0.05 was considered statistically significant. The average immunoglobulin (IG) dose was expressed as mean ± standard deviation. Statistical analysis was performed using XLSTAT Software (Addinsoft).

## Results

### Demographics and PID Distribution

Information on 2,728 PID patients was available for analysis. Of these patients, 1,851 (68%) were marked as alive and 200 (7%) as dead. The remaining 677 (25%) were not updated during the last year or were lost to follow-up. The male-to-female ratio was 1.5:1, with 1,657 male patients (60%) and 1,071 female (40%).

**Figure 1:**
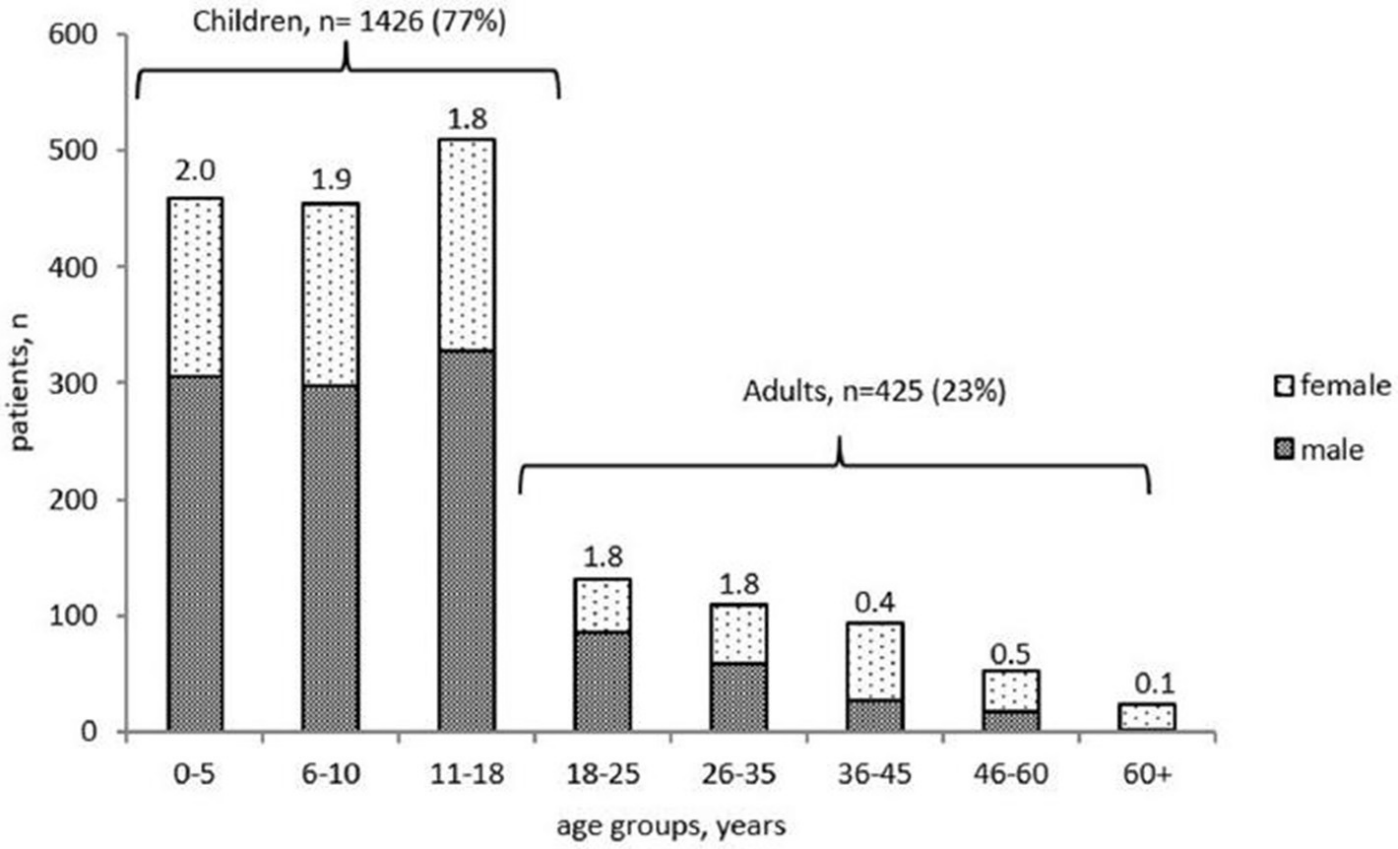
Age distribution and gender ratio of PID patients registered in the Russian PID registry (living 492 patients only, *n* = 1851).

Of the 1,851 living patients, 1,426 (77%) were children, and 425 (23%) were adults. The majority of the children (913 of 1,426, 64%) were under 10 years old. The male-to-female ratio varied from 2:1 in children, to 1:1 in the group of adults under the age of 30 and 0.4:1 in the older patients (Figure 1).

**Figure 2:**
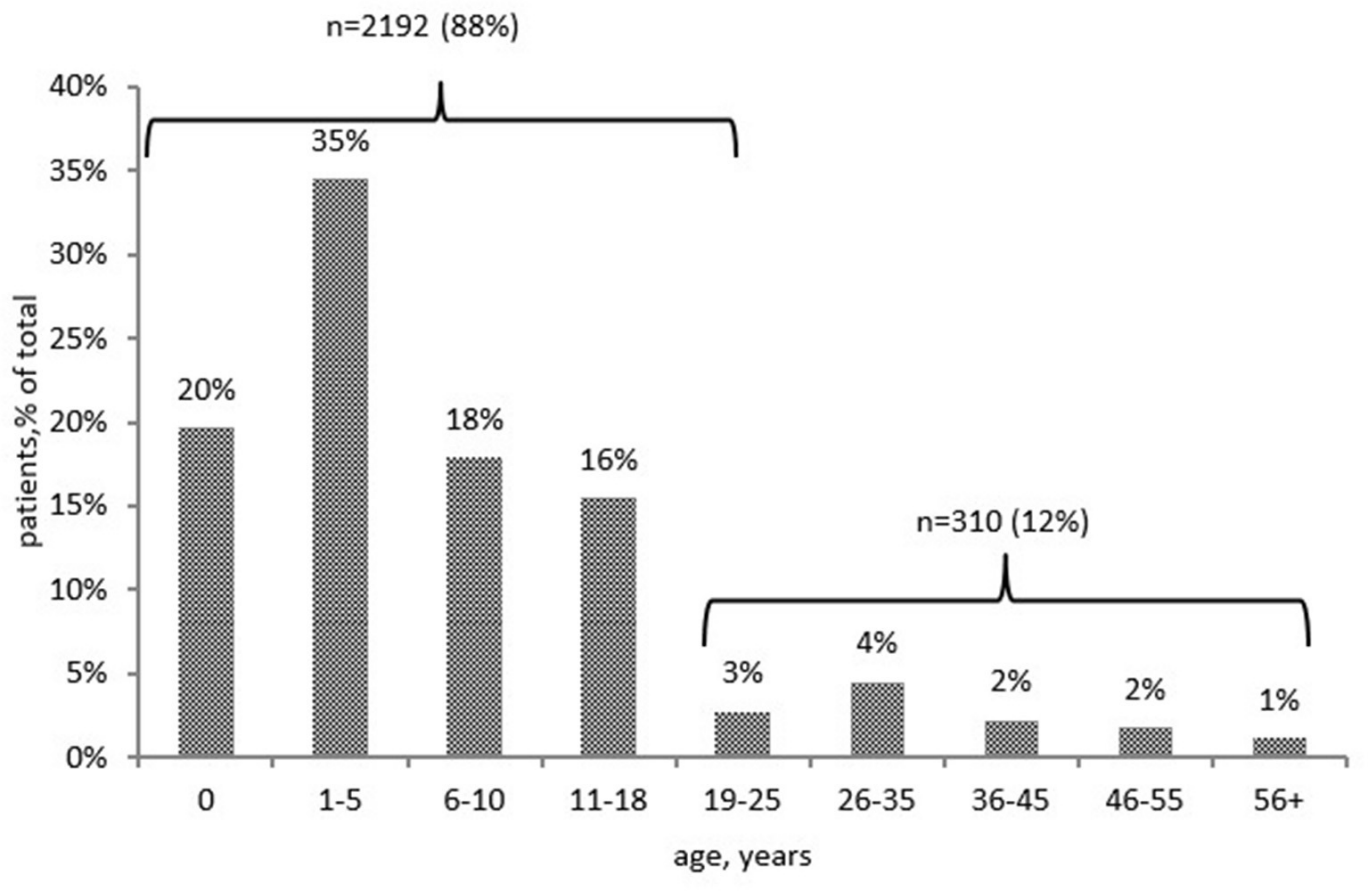
Patients' distribution by the age of PID diagnosis (*n* = 2,502).

PID was diagnosed before the age of 18 years (in childhood) in 2,192 patients (88%), predominantly in the first 5 years of life (1,356, 54%; Figure 2). The distribution of patients among the main PID groups varied greatly between children and adults. All forms of PID were observed in children and in young adults (under the age of 25 years). Yet the majority of older patients belonged to just two categories—common variable immunodeficiency (CVID) and hereditary angioedema (HAE).

**Figure 3:**
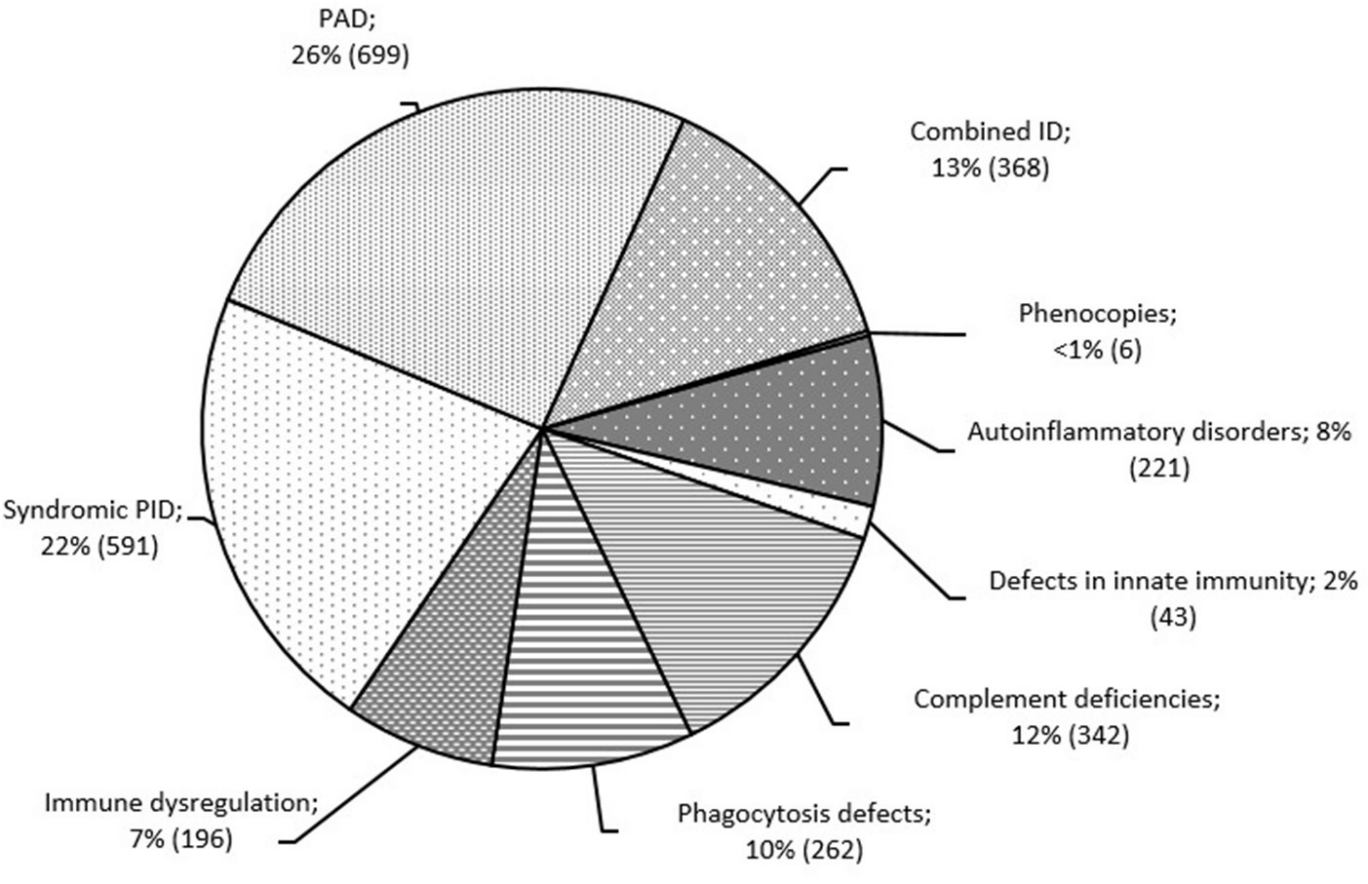
Distribution of patients among the PID groups (*n* = 2,728). PID groups are shown according to IUIS classification, 2017 (1). Total number of patients and percentage of all registered patients are shown for each group.

Overall, primary antibody deficiencies (PAD; 699; 26%) and syndromic PID (591; 22%) were the most common disorders in Russia. These were followed by five PID groups, in similar proportions: complement deficiencies (342; 12%), phagocytic defects (262; 10%), combined T and B cell defects (368; 13%), autoinflammatory disorders (221; 8%), and immune dysregulation (196; 7%; Figure 3). Somatic phenocopies (6; <1%) and defects of innate immunity (43; 1.5%) were very rare.

The most frequent PID categories in Russia, which cumulatively accounted for 53% of all registered patients, were: HAE type 1 and 2 (*n* = 341), CVID (*n* = 317), Wiskott–Aldrich syndrome (WAS; *n* = 154), X-linked agammaglobulinemia (XLA; *n* = 155), Chronic granulomatous disease (CGD; *n* = 135; of them 92 patients with X-linked CGD (X-CGD), Severe combined immunodeficiency (SCID; *n* = 137; of them 47 patients with X-linked SCID (X-SCID), DiGeorge syndrome (DGS; *n* = 130), Ataxia-telangiectasia (AT; *n* = 127) and Nijmegen breakage syndrome (NBS; *n* = 88; Table 1).

**Table 1:** Distribution of patients by PID groups [according to IUIS classification, 2017 (1)].

**PID Category**	**Patients, *n***	**Gender**		**Living status**			**Family cases**	**HSCT**
		**Female**	**Male**	**Alive**	**Deceased**	**Lost to follow-up**		
Autoinflammatory disorders	221	101	120	186	1	34	23	6
Defects in intrinsic and innate immunity	43	19	24	32	1	10	8	7
Complement deficiencies	342	215	127	210	3	138	99	–
Congenital defects of phagocyte number or function	262	71	191	186	14	62	41	66
Diseases of immune dysregulation	196	65	131	134	18	44	24	41
Combined immunodeficiencies with associated or syndromic features	591	214	377	405	59	127	57	106
Predominantly antibody deficiencies	699	238	461	453	21	225	36	5
Immunodeficiencies affecting cellular and humoral immunity	368	145	223	248	83	37	22	111
Phenocopies	6	3	3	6	–	–	–	–
Total number of patients	2,728	1,071	1,657	1,851	200	677	310	342

**Figure 4:**
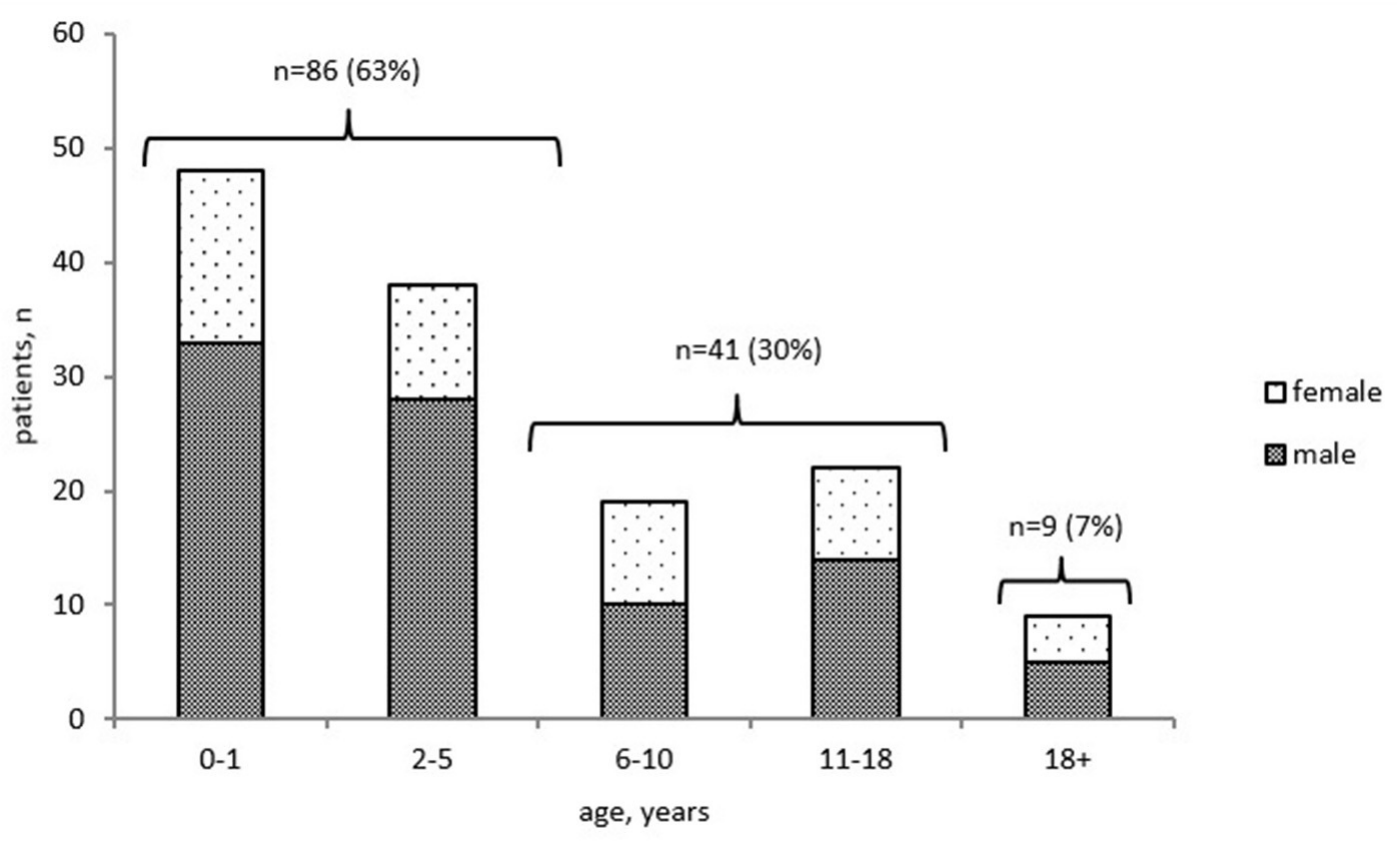
Distribution of deceased PID patients by age at death and gender (*n* = 136). Only patients with known date of death were included.

To assess mortality, we analyzed the cohort of 2,051 patients whose status was known (including 1,851 alive and 200 deceased patients). The overall mortality rate was estimated at 9.7%. The precise date of death was known for 136 of the 200 deceased patients: 127 (93%) children and 9 (7%) adults (Figure 4). The mortality rate ranged from 2 to 42% in different age groups; the highest rate was found in children in their first 2 years of life (Figure S1). The majority of infant deaths occurred in SCID patients (39 of 48, 81%; Figure S1). In the next age group (2–5 years), mortality was highest in the following four PID groups, in almost equal proportions: T and B cell defects (12/38, 32%) and syndromic PID (11/38, 29%), followed by phagocytic defects (7/38, 18%), and immune dysregulation (7/38, 18%). In total, 63% (86/136) of all PID-related deaths occurred in patients within the first 5 years of life. In older children (12–14)^1^, (15–17), mortality was associated predominantly with syndromic PID (55%), immune dysregulation (9%), and PAD (13%)—whereas, in adults, it was associated only with PAD (78%) and HAE (22%; Figure S1).

### Diagnostic Delay

**Figure 5:**
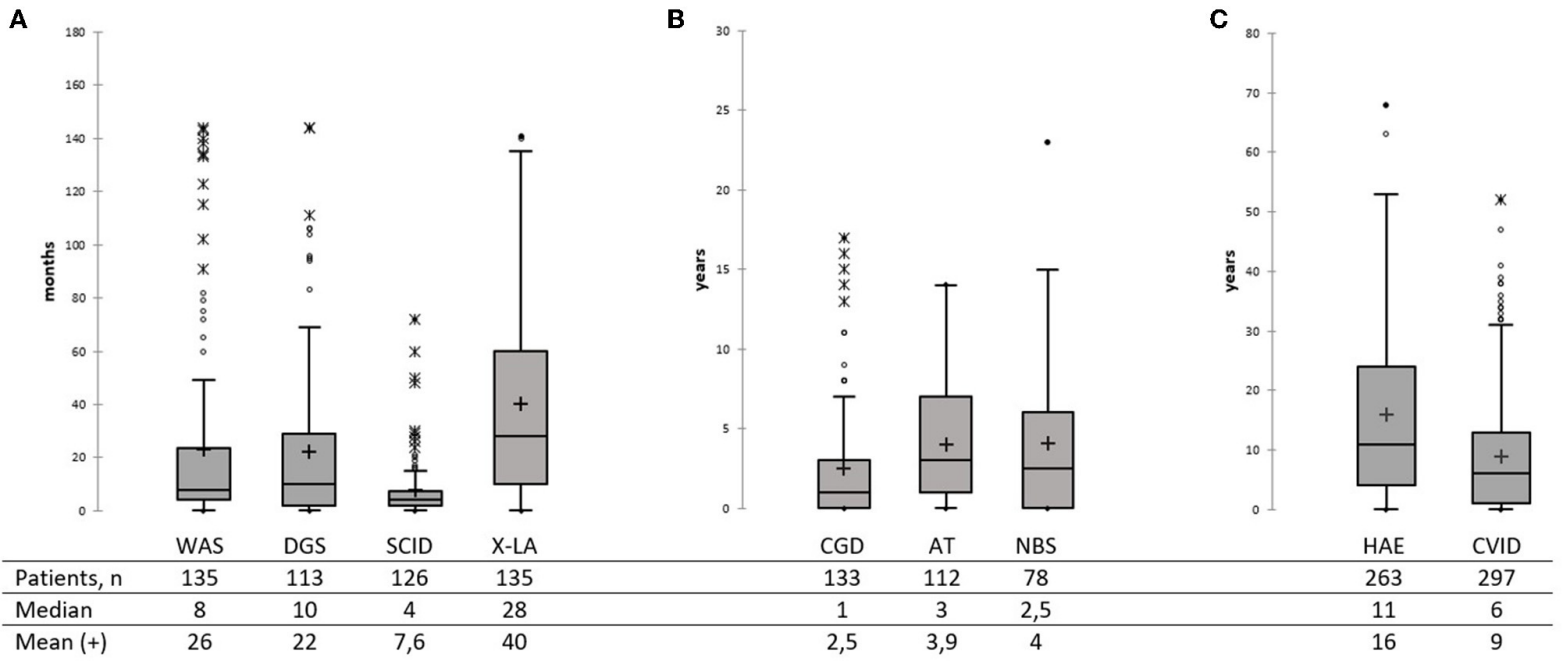
Diagnostic delay in the main PID categories **(A–C)**. Total numbers of patients, median, and mean are shown below the graph. Median is marked as a black line; mean is marked as a cross. **(A)** Diagnostic delay in WAS, SDG, SCID, and XLA patients. **(B)** Diagnostic delay in CGD, AT, and NBS patients. **(C)** Diagnostic delay in HAE and CVID patients.

Substantial PID diagnostic delay has been noted in Russia—with a median of 2 years for the whole group, but over a broad age range (0–68 years). No difference in diagnostic delay was observed, between patients diagnosed during the last 5 years (*M* = 2; 0–63, 997 patients) and before 2015 (*M* = 2 years; 0–68, 1,400 patients). Among the most common PID, the shortest diagnostic delay was observed in SCID (*M* = 4 months, 0–68), followed by the WAS (*M* = 8 months, 0–144), DGS (*M* = 10 months, 0–144), and CGD (*M* = 1, 0–17 years; Figure 5A). In X-linked agammaglobulinemia (XLA) patients, time to diagnosis varied greatly—from 0 to 141 months, with a median of 28 months. The DNA repair disorders NBS and AT were diagnosed with a median of 2.5 years (0–23) and 3.0 years (0–14), respectively (Figure 5B). The longest diagnostic delay was observed in CVID (*M* = 6 years, 0–52) and HAE (*M* = 11 years, 0–68; Figure 5C).

Just a few PID patients were diagnosed before the clinical onset of the disease, due to their family history; genetic testing was carried out for each of them. These included seven children with mutations in *SERPING1*, two with *BTK*, one with *WAS*, and one with *JAK3* defects. Genetic diagnosis led to an early start on IVIG therapy in the XLA patients, and to successful HSCT in the WAS and SCID patients.

### Family History

The registry contained 310/2,728 (11%) familial PID cases, originating from 150 families (Table 1), with the most frequent familial PIDs being HAE, WAS, and XLA. Consanguinity, as reported by the parents, was documented in 45 families. A family history of at least one death suspected to be due to PID was documented for 275 patients. These included infection-related deaths, in 185 cases, and malignancy-related deaths in 49 cases.

### Epidemiology

**Figure 6:**
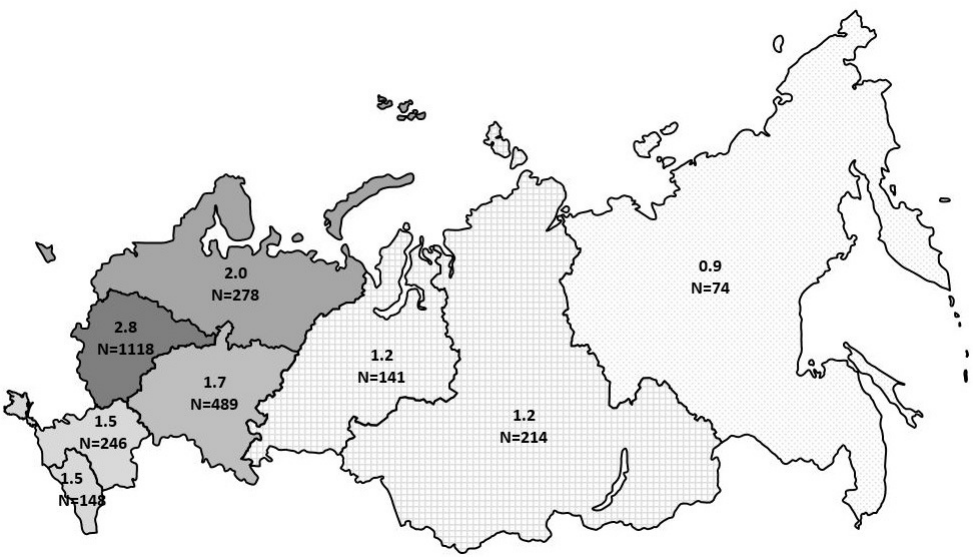
Prevalence of PID in Russia by federal district. The numbers represent prevalence per 100,000 people and total number of registered PID patients in each district. The registered number includes living and deceased patients.

**Figure 7:**
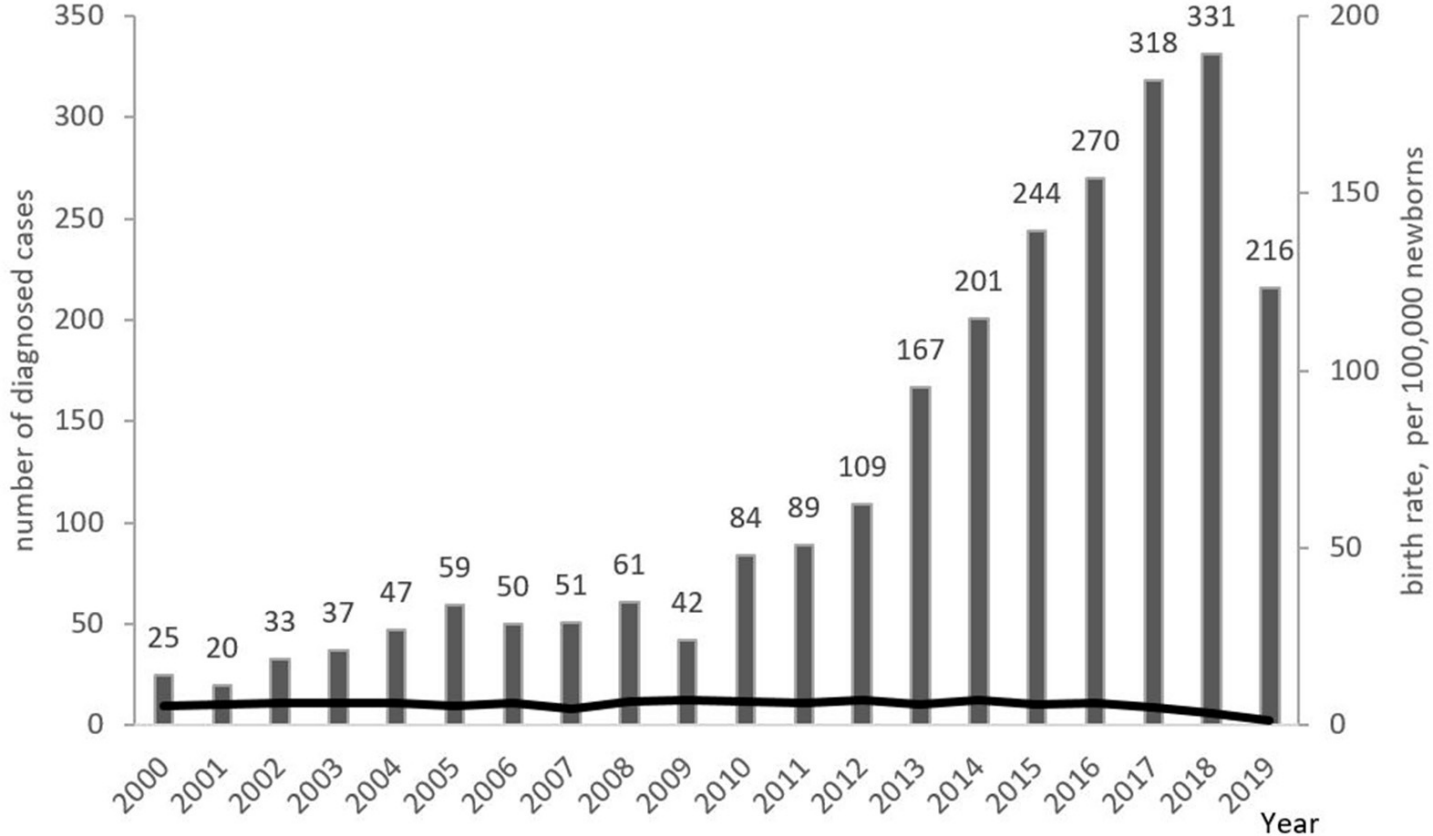
Annual PID incidence and numbers of newly diagnosed PID cases. Incidence is presented as the number of PID born each year per 100,000 newborns (shown as black line); newly diagnosed PID cases (columns) are presented as the number of patients registered in each year. Note that the lower number of patients in 2019 represents a lag in patients' registration into the database.

The minimum overall PID prevalence in the Russian population was estimated at 1.3:100,000 people, with drastic variations among the federal districts (from 0.9 to 2.8 per 100,000; Figure 6). The average annual PID incidence was estimated to be 5.7 ± 0.6 in 100,000 live births. This ranged from 4.4 to 7.1:100,000, over the period from 2000 to 2019. During this period, the average number of newly diagnosed PID cases per year increased from 201 to 331 (Figure 7).

Prevalence was estimated only for those PIDs frequently found in the adult group and with a low number of deaths registered in the database—CVID and HAE, with 0.22 and 0.23 per 100,000 people, respectively. This represents population frequency rates of 1 case per 430,000–450,000 people.

### Genetic Defects

Genetic testing has been performed for 1,740 patients, with genetic defects confirmed in 1,344 (77%). PID diagnosis has been genetically confirmed in 86% of the children, yet in only 12% of the adults.

Disease-causing genetic defects were detected by the following genetic methods: by direct Sanger sequencing in 903 patients (67%) and by next-generation sequencing (NGS) methods in 323 (24%) patients [including targeted panels, in 278; whole exome sequencing (WES), in 30; Clinical exome, in 13; and whole genome sequencing (WGS), in 2]. In the remaining 118 (9%) patients, cytogenetic methods and MLPA were used. Deletion of 22q.11 was confirmed via the FISH method in 80 patients, and by CMA in 26. In 6 cases, various chromosomal abnormalities resulting in syndromic forms of PID were confirmed by CMA.

Mutations were found in 98 PID genes and in three genes that are not currently included in the PID classification (*NTRK1, SCN9A, XRCC4*) (Table 2).

**Table 2:** Distribution of patients by individual disorders/genetic defects.

	**Patients, *n***
**PID Category**	**With genetic diagnosis/total (% of total)**
**Autoinflammatory disorders**	**109/221 (49%)**
***Defects affecting the inflammasome***	
MEFV	31
MVK	26
NLRP3	22
PSTPIP1	8
TNFRSF1A	8
polygenic: PSMB8, PSMA5, PSMC5	1
NLRP1	1
POMP	1
PLCG2	1
***Non-inflammasome-related conditions***	
IL36RN	1
***Type 1 interferonopathies***	
ADA2	3
IFIH1, GOF	3
TMEM173	2
ADAR1	1
**Defects in intrinsic and innate immunity**	**27/43 (63%)**
***Epidermodysplasia verruciformis (HPV)***	
CXCR4	9
***Mendelian susceptibility to mycobacterial disease (MSMD)***	
STAT1, AD LOF	1
STAT1, AR LOF	1
IL12RB1	3
IFNGR2	1
IFNGR1	1
***Predisposition to mucocutaneous candidiasis***	
STAT1, GOF, del3p25.3^*^	1
STAT1, GOF	6
***Predisposition to severe viral infection***	
STAT2	1
***Other inborn errors of immunity related to non-hematopoietic tissues***	
NBAS	2
TCIRG1	1
**Complement deficiencies**	**179/342 (52%)**
CFHR3	1
***HAE***	178/341
SERPING1	178
**Congenital defects of phagocyte number or function**	**185/262 (71%)**
***Congenital neuntropenias***	79/107 (74%)
ELANE	32
GFI1	1
G6PC3	1
SBDS	38
USB1	1
SMARCD2	1
CSF3R	1
Tafazzin (TAZ)	1
WAS, GOF	2
SLC37A4	1
***Defects of respiratory burst***	101/135 (75%)
CYBB	91
CYBB, 4XXY^*^	1
CYBA	6
NCF1	2
NCF2	1
***Other non-lymphoid defects***	
GATA2	3
***Defects of motility***	
ITGB2	1
RAC2	1
**Diseases of immune dysregulation**	**109/196 (56%)**
***Familial hemophagocytic lymphohistiocytosis (FHL syndromes)***	
PRF1	1
UNC13D	8
STXBP2	4
***FHL syndromes with hypopigmentation***	
LYST	2
RAB27A	1
***Regulatory T cell defects***	
FOXP3	8
CTLA4	11
CTLA4, del2q.33.2^*^	1
LRBA	2
STAT3, GOF	4
***Autoimmunity with or without lymphoproliferation***	
AIRE	11
***Autoimmune lymphoproliferative syndrome***	
CASP10	2
FAS	25
***Immune dysregulation with colitis***	
IL10RA	1
***Susceptibility to EBV and lymphoproliferative conditions***	
XIAP/ BIRC4	16
SH2D1A	10
RLTPR	2
**Combined immunodeficiencies with associated or syndromic features**	**457/591 (77%)**
***Immunodeficiency with congenital thrombocytopenia***	
WAS	154
***Anhidrotic ectodermodysplasia with immunodeficiency***	
IKBKG	3
IKBA	1
***DNA repair defects***	
NBN	75
ATM	53
polygenic: ATM, NFKB1	1
ATM, dup4p16.3^*^	1
BLM (RECQL3)	2
DNMT3B (ICF1)	2
MRE11	1
ZBTB24 (ICF2)	2
***Thymic defects with additional congenital anomalies***	
22q11.2DS	106
CHD7	1
SEMA3E	1
***Immuno-osseous dysplasias***	
SMARCAL1	5
RMRP	4
***Hyper IgE syndromes (HIES)***	
STAT3, LOF	21
SPINK5	4
***Other defects***	
CCBE1	1
KMT2D	15
***Chromosomal microdeletions***	
10p.13-10p.14DS	1
11q23del	1
psu dic (21;Y)(q22;q11.1); 21q11.1, 21q21.1-q22.12, 21q22.3 (including IL10RB, IFNAR2)	1
46XX-21	1
11q13.5-q23.1	1
**Predominantly antibody deficiencies**	**144/699 (21%)**
***X-LA***	114/155 (74%)
BTK	113
BTK, del11p^*^	1
NFKB1	6
NFKB1, del 4q22.3-q25^*^	1
NFKB2	1
PIK3CD, GOF	8
PIK3R1, GOF	5
AICDA	1
TCF3	2
TNFRSF13B (TACI)	5
TRNT1	1
**Immunodeficiencies affecting cellular and humoral immunity**	**127/368 (35%)**
***SCID***	92/137 (67%)
**T-B- SCID**	
RAG1	16
RAG2	4
ADA	6
ARTEMIS	8
***T-B+SCID***	
IL2RG	44
IL7RA	2
JAK3	6
LIG4	4
NHEJ1	1
CORO1A	1
***Combined immunodeficiency (CID), generally less profound than SCID***	31
PNP	1
CARD11	1
CD40LG	24
DOCK2	1
DOCK8	3
RFXANK	1
***Not classified***	
NTRK1	1
SCN9A	2
XRCC4	1
**Phenocopies**	**6**
KRAS	5
NRAS	1

* *Patients with complex phenotype; GOF, gain-of-function variant; LOF, loss-of-function variant*.

As expected, the highest number of genetic defects were found in genes underlying the most frequent “classical” PID: mutations in *SERPING1* were found in 178 of 341 HAE cases (52.2%), WAS in 154 (100%) of WAS patients*, BTK* in 114 of 155 X-LA (73.5%)*, CYBB* in 98 (73%) of CGD 135 cases*, NBN* in 75/88 (85%) of NBS patients and *ATM* in 55/127 (43%) of AT patients. 106/130 DGS patients had del22q.11 confirmed. At least 20 patients (for each disease) had mutations in the following genes: *MEFV, MVK, NLRP3, ELANE, SBDS, FAS, STAT3 LOF, IL2RG*, and *CD40LG*. Rare defects, with 4–20 patients for each gene, affected predominantly recently described genes: *PSTPIP1, TNFRSF1A, CXCR4, STAT1, CYBA, STXBP2, FOXP3, CTLA4, AIRE, XIAP, SH2D1A, SMARCAL1, RMRP, SPINK5, KMT2D, NFKB1, PIK3CD, PIK3R1, TNFRSF13B, RAG1, RAG2, ADA, ARTEMIS, JAK3, LIG4*, and *KRAS*. The remaining 57 genes had mutations recorded for single (1–3) patients (Table 2).

The proportion of patients with genetically confirmed diagnoses was highest among those with syndromic PIDs, reaching 77% (457/591) (Table 1). Within the phagocytic defect and innate immunity defect groups, 71% (185/262) and 63% (27/43) of the patients, respectively, had a genetic diagnosis. PID genetic confirmation showed about half of all patients in the groups to have immune dysregulation (56%; 109/196), autoinflammatory disorders (49%; 109/221), and complement deficiencies (52%; 179/342)—the last of these due mainly to HAE. The proportion of patients with genetic diagnoses showing T- and B-cell defects was 33% (123/368). The lowest number of patients with verified mutations, at 21% (144/699), was observed in the PAD group (Table 1); BTK abnormalities prevailed among them (114/155; 73.5%).

Somatic mutations in KRAS and NRAS were confirmed in six patients.

The segregation of genetic defects by mode of inheritance was nearly equal: 469 patients (38.4%) with an X-linked (XL) diseases had mutations in 10 genes, 383 (31.4%) patients with autosomal dominant (AD) diseases had mutations in 29 genes, and 369 (30.2%) patients with autosomal recessive (AR) diseases had mutations in 58 genes.

**Figure 8:**
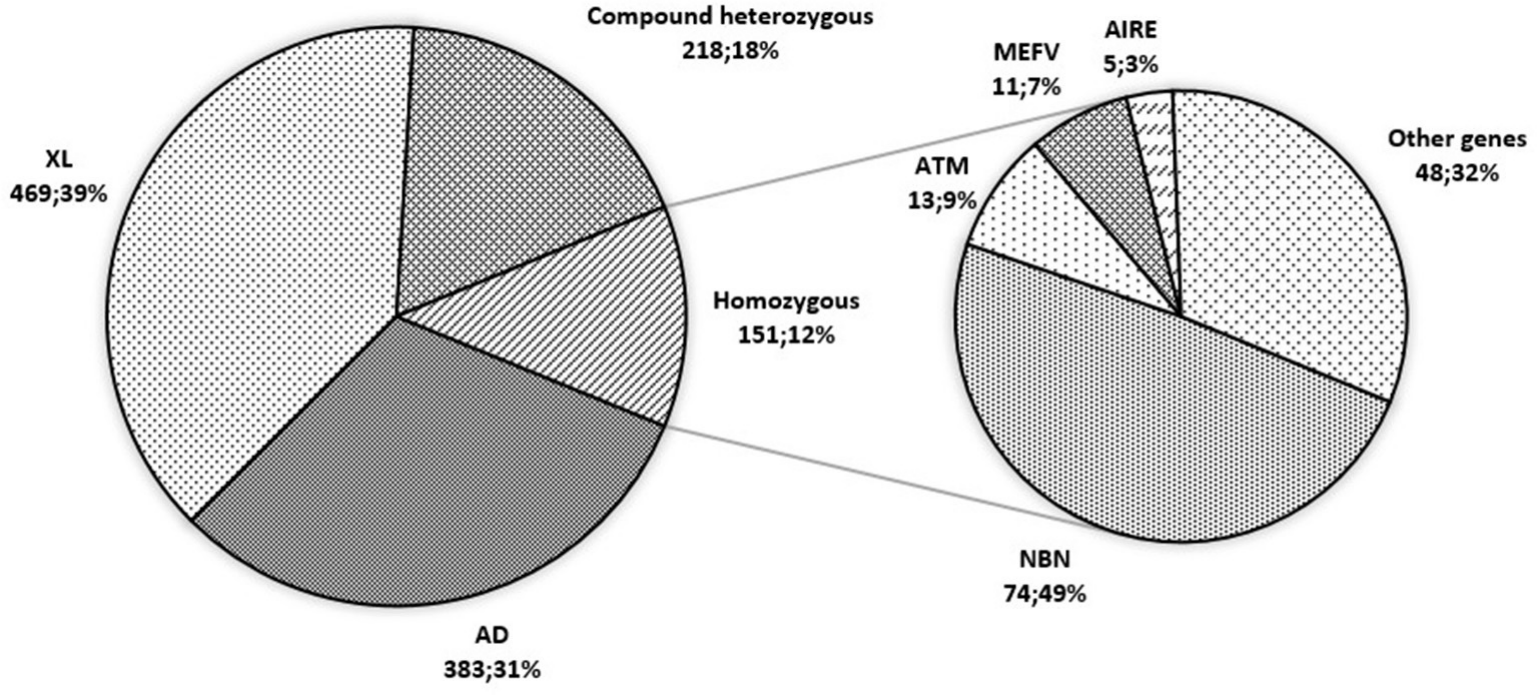
Inheritance of PID in Russia and most frequent genes with homozygous mutations.

In the group of AR PID patients, 218 (59%) had compound heterozygous mutations and 151 (41%) had homozygous mutations; the majority (74; 49%), as expected, were NBS patients with the “Slavic” mutation in the *NBN* gene (Figure 8). Homozygous mutations were also found in the genes with the known “hot-spots”: *MEFV* (11; 7%) and *AIRE* (5; 3%). Another “Slavic” mutation—*RAG1* c.256_257delAA p.K86fs, in a compound heterozygous or homozygous state—was reported in 7/16 patients with *RAG1* defects, putting this allele frequency at 25%.

Testing for prenatal PID diagnosis (PND) was performed in 40 pregnancies among 37 families with previously known PID-causing genetic defects. Embryonic/fetal material was obtained by chorionic villi sampling at 10–12 weeks of gestation in 37 cases; by amniocentesis in the second trimester, in two cases; and by cordocentesis, in one. No serious complications were noted, during or after the procedures. 30/40 embryos were mutation-free. In six cases, a PID diagnosis was given; all families chose to terminate the pregnancies. Four embryos were heterozygous carriers of recessive PID mutations—all these pregnancies were carried to term. Two more sibling heterozygous carriers were born after preimplantation diagnosis.

### Symptomatic Treatment

Treatment of PID symptoms, as documented in the registry, has been divided into three categories: immunoglobulin (IG) substitution, biologicals, and “other.” There was updated information for 1,622 patients, regarding prescribed or on-going therapy. Half of the patients (843/1,622, 52%) received IG substitution. Of these, only 32 patients (4%) have ever had an experience with subcutaneous IG (SCIG); all others received intravenous IG (IVIG), with an average dose of 0.46 ± 0.09 g/kg per month. Regular IG substitution therapy was recorded in 279/369 patients (76%) with syndromic PID, in 296/433 (68%) PAD patients and in 173/270 patients (64%) with combined PID. At least single (but not regular) IG use was recorded for 15/29 patients (52%) with defects of innate immunity, 61/124 patients (49%) with immune dysregulation, 49/172 patients (28%) with phagocytosis defects, and 25/171 patients (15%) with autoinflammatory disorders.

414/1,622 (25%) patients were treated with various biological drugs. Updated information was available for 91 HAE patients, of whom 70/91 (77%) received either a C1 inhibitor or a selective antagonist of bradykinin receptors during attacks, including 51 patients who had experience with both drugs. In other PIDs, the rate of biological treatment was highest in the group of patients with autoinflammatory disorders: 86/186 (46%). This was followed by the group of immune dysregulation, with 48/134 (36%); and of combined PID, with and without syndromic features: 63/405 (16%) and 27/242 (18%), respectively. Patients with disorders of innate immunity and PAD were treated with biologicals only, in 3/32 (9%) and in 43/453 (6%) cases, respectively.

### Curative Therapies

Three patients in the cohort underwent gene therapy for WAS; all are currently alive.

Information was available for 342/2,728 (16%) patients who underwent HSCT. Of these, 60 were deceased, 228 alive and 54 had not been updated during the prior 2 years (Table 1). All transplanted patients were diagnosed with PID as children. Yet, in 5/342, HSCT was performed after 18 years of age. HSCT has been performed in 106/591 (18%) patients with PIDs with syndromic features (18% of all syndromic PIDs), including 92/106 (88%) with WAS and 25/88(28%) with NBS; in 111 patients with combined T- and B-cell defects (30% of all CID), including 79/137 SCID (58%); in 66/262(25%) patients with phagocytic defects, including 47/135 CGD (35%) and 14/107 SCN (13%); in 41/196 (21%) patients with immune dysregulation; in 5/699 (0.7%) patients with PAD [four with activated PI3K syndrome (APDS) and 1 with XLA]; in 6/221(3%) patients with autoinflammatory disorders; and in 7/43 (16%) patients with defects of innate immunity.

## Discussion

The current study represents the first attempt to systematically assess clinical and epidemiological data on patients with PID in Russia, using the online registry.

At the time of analysis, 2,728 PID patients were registered, representing all districts of the country—thus making this study a valid assessment of the PID cohort in Russia. Since reporting patients in the registry was not mandatory for the treating physicians, we expect underreporting of about 15–20% and are therefore able to discuss only the minimum epidemiological characteristics of PID in Russia. Though PID prevalence in the central part of Russia (2.8 per 100,000 people) is comparable to that of most European countries (2–8 per 100,000 people) (4–6, 15–17), the overall prevalence of 1.3 per 100,000 is quite low. This reflects significant under-diagnosis, especially in regions with low population density and economic status.

The male-to-female ratio in our various age groups does not differ greatly from previous observations, with males predominating amongst children and females in adulthood (4, 16, 17) (Figure 1).

Our study demonstrates a high mortality rate in the Russian PID cohort—as high as 9.8%—as compared with the most recently published German and Swiss registry. Yet it is comparable to the 8.6% (641/7,430) in the previously published ESID registry (18) and the 8% (2,232/27,550) provided by the online ESID reporting website^2^. Significantly, half of reported PID deaths occur within the first 5 years of life. This stresses the importance of early PID diagnosis and quick referral to transplanting centers, as SCID and other CIDs account for the majority of early PID deaths. In light of these statistics, unrecognized infant PID mortality may significantly contribute to the low prevalence of PID in Russia, as patients die before they are diagnosed with PID. Thus, future introduction of neonatal PID screening utilizing TREC/KREC detection may substantially improve PID verification (19, 20).

Children represent the majority (77%) of PID patients in the registry. Comparing this data to other registries—where patients over 18 years old represent up to 55% of all PID (4),^2^ (18)—we can conclude that adults with PID are the most under-diagnosed category in Russia. This statement is confirmed by the low proportion of PAD defects in the Russian registry (21 vs. 56% in the ESID registry) (9),^2^ (18, 21). This, in turn, reflects low numbers of patients with CVID, the main PID affecting adults worldwide. The estimated prevalence of CVID in Russia is 0.2 per 100,000 people—whereas, in other registries, CVID prevalence reaches 1.3 per 100,000 people (22).

In the recent years, Russia developed a relatively good network of pediatric immunologists, yet adult immunologists are scarce. NAEPID and the registry team have an educational and organizational plan aimed at improving adult PID diagnosis and care. The registry will be a good tool to assess success of the project in the next 5 years.

Combined immunodeficiencies with syndromic features constitute the most prominent PID group in the registry (22%), presumably due to the well-defined phenotype and the high awareness of these disorders among various medical specialists. Patients with WAS and DGS have the shortest diagnostic delay and the highest proportion of genetic confirmation. Overall, the majority of genetic defects were confirmed in the clinically or analytically well-defined and well-described PID (HAE, WAS, XLA, CGD, and NBS). Most studies also have the highest genetic confirmation rate in the group of combined PID (4, 6), though an Iranian study describes a predominance of genetic defects in the dysregulation group (17).

The patients' distribution amongst PID groups differs from that of most published registries in other aspects, as well. Though PAD are underrepresented, we have relatively large groups of autoinflammatory disorders (AID) and complement defects (predominantly HAE). This is because the Russian PID database collects data on all IUIS classified PIDs, in contrast with some other countries—where AID cases are followed and reported predominantly by rheumatologists, and HAE cases predominantly by allergists (23, 24). In our registry, HAE patients contribute 12% of all PID cases and have a high rate of genetic confirmation, though diagnostic delay in these cases is still quite high.

Overall, diagnostic delay amongst the predominant forms of PID varied from 4 months in SCID—which is similar to data reported by others (22, 25)—to 141 months in XLA patients. Obviously, such long diagnostic delays lead to a number of unrecognized PAD deaths and contribute to the low proportion of humoral deficiencies in the registry.

Diagnostic delay amongst NBS patients was shorter (median 2.5 years) than that reported previously in a smaller cohort of Russian NBS patients (median 5.0 years) (11). Yet the range of diagnostic delay is rather substantial: some patients were diagnosed as teenagers only after the onset of a malignancy, in spite of continuous follow-up by neurologists.

Sadly, with the increase of PID diagnosis in the last 5 years, there has been no improvement in diagnostic delay. This, yet again, raises the question of neonatal screening. Wider availability of next-generation sequencing methods, which were routinely introduced in Russia only in 2017, may also change this dynamic.

Unsurprisingly, 67% of the genetic defects in our cohort were detected via Sanger sequencing, in the most frequent and well-defined PID (2). A significant proportion of the mutations in the recently described genes were confirmed only with the advent of NGS techniques (26–29). NGS has allowed us to detect mutations in as many as 80 PID genes, sometimes with only one or a few patients per gene. The application of NGS to PID diagnosis has revolutionized the field by identifying novel disease-causing genes and allowing for the quick and relatively inexpensive detection of defects therein (27, 29). Adult PIDs show a substantially lower rate of genetic confirmation than that seen in children. This is partially because genetic defects are often not found in CVID, even using NGS methods (30, 31). Yet it also represents the fact that adults are less likely to pay for genetic testing since, in Russia, it is not covered by the state or by medical insurance.

As described by others (4, 6) the majority of the genetic defects were found in males, due to the fact that a lot of the “old” PID have X-linked inheritance.

In highly consanguineous populations, AR PIDs represent 70–90% of cases (32, 33). Interestingly—though the Russian population is very heterogeneous, with low numbers of consanguineous marriages (45 families, 1.9%)—AR genetic defects comprised 30% of all defects described in the cohort, with 40% of these being homozygous for the respective mutations. This is due to the “founder effect,” known for affecting several PID genes in the Slavic population. The majority of NBS patients−74 (98.7%)—were homozygous for the “Slavic” mutation (11). A high frequency of the RAG1 c.256_257delAA p.K86fs mutation is also typical for Slavic populations, as previously noted (34).

Other homozygous mutations were reported in patients with defects in the *MEFV* and *AIRE* genes, and known for the hot-spot mutations. (35–39).

Our cohort included a group of patients with large aberrations, involving at least one PID gene. Therefore, we conclude that patients with complex phenotypes require implementation of not just Sanger sequencing and/or NGS methods, which can only indirectly point to a large aberration, but also cytogenetic methods, including CMA. Moreover, even well-described PIDs like HAE often require a combination of genetic methods, including MLPA, to detect large deletions frequent in this disease (2).

Our first analysis of the Russian PID population demonstrates substantial genetic diversity and high rate of genetic diagnosis confirmation-−49% of all registered patients. This is comparable to 36–43% of genetic PID confirmation in patients from French and German registries (4, 6).

The importance of genetic defect verification cannot be underestimated, as it influences overall treatment approach (HSCT vs. conservative treatment) and targeted therapy validation. It is also crucial for prenatal/preimplantation testing—which, if implemented, allows families to have healthy children. This is especially important for families with currently incurable PIDs, like AT and some others.

As previously published (40), the main treatment strategy for most PID patients (52% in the current study) is regular IG replacement. Additionally—in contrast to European data (4, 16)—the vast majority of patients in Russia are treated with IVIG, with only 4% of the patients having experience with subcutaneous IG replacement. Hence, IG substitution in Russia requires systemic modifications, i.e., wider availability of SCIG and home IVIG infusions that are not available at this time.

To our knowledge, the Russian PID registry is the first to analyze the use of monoclonal antibodies and other biologics in the treatment of PID symptoms. The number of patients treated with this kind of therapy in this cohort is rather high, reaching 25%.

Finally, 12% of patients underwent curative treatment, predominantly HSCT—a number comparable to the German registry (4). The proportions of transplanted patients with phagocytic disorders and with immune dysregulation were also similar in both registries. Yet, in comparison with the German registry—where one third of all HSCT was performed in CID patients—the predominant HSCT group in Russia consisted of patients with syndromic PIDs (18%) This reflects a significant prevalence of NBS patients, for whom HSCT has shown to be a successful and safe treatment strategy (11).

In conclusion, the current study has summarized the epidemiological features of PID patients in Russia and highlighted the main challenges for the diagnosis and treatment of patients with PID. As with all other rare disease registries, the Russian PID registry is a powerful tool—not just for data collection but also to help improve PID patient care, especially in the setting of a large country with highly diverse regional features.

## Data Availability Statement

The raw data supporting the conclusions of this article will be made available by the authors, without undue reservation.

## Ethics Statement

This study was approved by the ethics committee of the Dmitry Rogachev National 474 Center of Pediatric Hematology, Oncology, and Immunology (approval No 2∋/2-20). All patients or their 475 legal guardians gave written informed consent for participation in the registry.

## Author Contributions

All authors listed have made a substantial, direct and intellectual contribution to the work, and approved it for publication.

## Conflict of Interest

The authors declare that the research was conducted in the absence of any commercial or financial relationships that could be construed as a potential conflict of interest.

## Footnotes

^1^Available online at: https://showdata.gks.ru/finder/descriptors/274848 (accessed March 1, 2020).

^2^Available online at: https://cci-reporting.uniklinik-freiburg.de/#/ (accessed March 1, 2020).

## Supplementary Material

Figure S1 Mortality rate by PID group and age of the deceased patients. Mortality rate in each age group is shown on the top of the stacked bars as a ratio of deceased patients to all patients of the relevant age. Textures represent different PID groups. Numbers next to the plots show the number of patients and the ratio of the deceased patients by PID group to the total number of the deceased patients of the age group.

## References

[1] Picard C Bobby Gaspar H Al-Herz W Al-Herz W Bousfiha A Casanova JL. International Union of Immunological Societies: 2017 Primary immunodeficiency diseases committee report on inborn errors of immunity. J Clin Immunol. (2018) 38:96–128. 10.1007/s10875-017-0464-9 29226302 PMC5742601

[2] Heimall JR Hagin D Hajjar J Henrickson SE Hernandez-Trujillo HS Tan Y Use of genetic testing for primary immunodeficiency patients. J Clin Immunol. (2018) 38:320–9. 10.1007/s10875-018-0489-8 29675737

[3] Leiding JW Forbes LR. Mechanism-based precision therapy for the treatment of primary immunodeficiency and primary immunodysregulatory diseases. J Allergy Clin Immunol Pract. (2019) 7:761–73. 10.1016/j.jaip.2018.12.017 30832891

[4] El-Helou SM Biegner AK Bode S Ehl SR Heeg M Maccari ME. The German National registry of primary immunodeficiencies (2012-2017). Front Immunol. (2019) 10:1272. 10.3389/fimmu.2019.01272 31379802 PMC6659583

[5] Marschall K Hoernes M Bitzenhofer-Grüber M Jandus P Duppenthaler A Wuillemin WA. The Swiss National Registry for Primary Immunodeficiencies: report on the first 6 years' activity from 2008 to (2014). Clin Exp Immunol. (2015) 182:45–50. 10.1111/cei.12661 26031847 PMC4578507

[6] Mahlaoui N Picard C Bach PB Costes LB Courteille VB Ranohavimparany A. Genetic diagnosis of primary immunodeficiencies: a survey of the French national registry. J Allergy Clin Immunol. (2019) 143:1646–49.e10. 10.1016/j.jaci.2018.12.994 30639347

[7] Mahlaoui N Jais JP Brosselin P Mignot C Beaurain B Brito C. Prevalence of primary immunodeficiencies in France is underestimated. J Allergy Clin Immunol. (2017) 140:1731–3. 10.1016/j.jaci.2017.06.020 28732644

[8] Kerner G Ramirez-Alejo N Seeleuthner Y Yang R Ogishi M Cobat A. Homozygosity for TYK2 P1104A underlies tuberculosis in about 1% of patients in a cohort of European ancestry. Proc Natl Acad Sci U S A. (2019) 116:10430–4. 10.1073/pnas.1903561116 31068474 PMC6534977

[9] Grimbacher B on behalf of the ESID Registry Working Party. The European Society for Immunodeficiencies (ESID) registry 2014. Clin Exp Immunol. (2014) 178:18–20. 10.1111/cei.12496 25546747 PMC4285476

[10] Kindle G Gathmann B Grimbacher B. The use of databases in primary immunodeficiencies. Curr Opin Allergy Clin Immunol. (2014) 14:501–8. 10.1097/ACI.0000000000000113 25225780

[11] Deripapa E Balashov D Rodina Y Laberko A Myakova N Davydova NV. Prospective study of a cohort of Russian Nijmegen breakage syndrome patients demonstrating predictive value of low kappa-deleting recombination excision circle (KREC) numbers and beneficial effect of hematopoietic stem cell transplantation (HSCT). Front Immunol. (2017) 8:807. 10.3389/fimmu.2017.00807 28791007 PMC5523727

[12] Laberko A Sultanova E Gutovskaya E Shipitsina I Shelikhova L Kurnikova E. Mismatched related vs matched unrelated donors in TCRαβ/CD19-depleted HSCT for primary immunodeficiencies. Blood. (2019) 134:1755–63. 10.1182/blood.2019001757 31558465 PMC6856988

[13] Tangye SG Al-Herz W Bousfiha A Chatila T Cunningham-Rundles C Etzioni A Human inborn errors of immunity: 2019 Update on the Classification from the International Union of Immunological Societies Expert Committee. J Clin Immunol. (2020) 40:24–64. 10.1007/s10875-019-00737-x 31953710 PMC7082301

[14] European Society for Immunodeficiencies Registry Working Party Diagnosis Criteria. (2018). Available online at: https://esid.org/Working-Parties/Registry-Working-Party/Diagnosis-criteria (accessed December 3, 2019).

[15] Ludviksson BR Sigurdardottir ST Johannsson JH Haraldsson A Hardarson TO. Epidemiology of primary immunodeficiency in Iceland. J Clin Immunol. (2015) 35:75–9. 10.1007/s10875-014-0107-3 25315263

[16] Shillitoe B Bangs C Guzman D Gennery AR Longhurst HJ Slatter M. The United Kingdom Primary Immune Deficiency (UKPID) registry 2012 to 2017. Clin Exp Immunol. (2018) 192:284–91. 10.1111/cei.13125 29878323 PMC5980391

[17] Abolhassani H Kiaee F Tavakol M Chavoshzadeh Z Mahdaviani SA. Fourth update on the Iranian National Registry of primary immunodeficiencies: integration of molecular diagnosis. J Clin Immunol. (2018) 38:816–32. 10.1007/s10875-018-0556-1 30302726

[18] Gathmann B Grimbacher B Beauté J Dudoit Y Mahlaoui N. The European internet-based patient and research database for primary immunodeficiencies: results 2006-2008. Clin Exp Immunol. (2009) 157:3–11. 10.1111/j.1365-2249.2009.03954.x 19630863 PMC2715433

[19] Brown L Xu-Bayford J Allwood Z Slatter M Cant A Davies E. Neonatal diagnosis of severe combined immunodeficiency leads to significantly improved survival outcome: the case for newborn screening. Blood. (2011) 117:3243–6. 10.1182/blood-2010-08-300384 21273302

[20] Thomas C Durand-Zaleski I Frenkiel J Mirallié S Léger A Cheillan D. Clinical and economic aspects of newborn screening for severe combined immunodeficiency: DEPISTREC study results. Immunology. (2019) 202:33–9. 10.1016/j.clim.2019.03.012 30946917

[21] Kobrynski L Powell RW Bowen S. Prevalence and morbidity of primary immunodeficiency diseases, United States 2001-2007. J Clin Immunol. (2014) 34:954–61. 10.1007/s10875-014-0102-8 25257253 PMC4820073

[22] Edgar JDM Buckland M Guzman D Conlon NP Knerr V Bangs C. UKPID registry 2008-2012. Clin Exp Immunol. (2014) 175:68–78. 10.1111/cei.12172 23841717 PMC3898556

[23] Betschel S Badiou J Binkley K Borici-Mazi R Hébert J Kanani A. The International/Canadian Hereditary Angioedema Guideline. Allergy Asthma Clin Immunol. (2019) 15:72. 10.1186/s13223-019-0376-8 31788005 PMC6878678

[24] Toplak N Frenkel J Ozen S Lachmann H Woo P Paut I. An international registry on autoinflammatory diseases: the eurofever experience. Ann Rheum Dis. (2012) 71:1177–82. 10.1136/annrheumdis-2011-200549 22377804

[25] Cirillo E Cancrini C Azzari C Martino S Martire B Pession A. Clinical, immunological, and molecular features of typical and atypical severe combined immunodeficiency: report of the italian primary immunodeficiency network. Front Immunol. (2019) 10:1908 10.3389/fimmu.2019.01908 31456805 PMC6700292

[26] Maffucci P Filion CA Boisson B Itan Y Shang L Casanova JL. Genetic diagnosis using whole exome sequencing in common variable immunodeficiency. Front Immunol. (2016) 7:220. 10.3389/fimmu.2016.00220 27379089 PMC4903998

[27] Seleman M Hoyos-Bachiloglu R Geha RS Chou J. Uses of Next-generation sequencing technologies for the diagnosis of primary immunodeficiencies. Front Immunol. (2017) 8:847. 10.3389/fimmu.2017.00847 28791010 PMC5522848

[28] Donadieu J Beaupain B Fenneteau O Bellanné-Chantelot C. Congenital neutropenia in the era of genomics: classification, diagnosis, and natural history. Br J Haematol. (2017) 179:557–74. 10.1111/bjh.14887 28875503

[29] Seidel MG Kindle G Gathmann B Quinti I Buckland M van Montfrans J. The European Society for Immunodeficiencies (ESID) registry working definitions for the clinical diagnosis of inborn errors of immunity. J Allergy Clin Immunol Pract. (2019) 7:1763–70. 10.1016/j.jaip.2019.02.004 30776527

[30] de Valles-Ibáñez G Esteve-Solé A Piquer M González-Navarro EA Hernandez-Rodriguez J Laayouni H. Evaluating the genetics of common variable immunodeficiency: monogenetic model and beyond. Front Immunol. (2018) 9:636. 10.3389/fimmu.2018.00636 29867916 PMC5960686

[31] Odnoletkova I Kindle G Quinti I Grimbacher B Knerr V Gathmann B. The burden of common variable immunodeficiency disorders: a retrospective analysis of the European Society for Immunodeficiency (ESID) registry data. Orphanet J Rare Dis. (2018) 13:201 10.1186/s13023-018-0941-0 30419968 PMC6233554

[32] Sheikhbahaei S Sherkat R Roos D Yaran M Najafi S Emami A. Gene mutations responsible for primary immunodeficiency disorders: a report from the first primary immunodeficiency biobank in Iran. Allergy Asthma Clin Immunol. (2016) 12:62. 10.1186/s13223-016-0166-5 27980538 PMC5133745

[33] Al-Herz W Chou J Delmonte OM Massaad MJ Bainter W Castagnoli R. Comprehensive genetic results for primary immunodeficiency disorders in a highly consanguineous population. Front Immunol. (2019) 9:3146. 10.3389/fimmu.2018.03146 30697212 PMC6340972

[34] Sharapova SO Guryanova IE Pashchenko OE Kondratenko IV Kostyuchenko LV. Molecular characteristics, clinical and immunologic manifestations of 11 children with omenn syndrome in East Slavs (Russia, Belarus, Ukraine). J Clin Immunol. (2016) 36:46–55. 10.1007/s10875-015-0216-7 26596586

[35] Jalkh N Génin E Chouery E Delague V Medlej-Hashim M Idrac CA. Familial Mediterranean fever in Lebanon: founder effects for different MEFV mutations. Ann Hum Genet. (2008) 72:41–7. 10.1111/j.1469-1809.2007.00386.x 17711558

[36] Cekin N Akyurek ME Pinarbasi E Ozen F. MEFV mutations and their relation to major clinical symptoms of Familial Mediterranean fever. Gene. (2017) 626:9–13. 10.1016/j.gene.2017.05.013 28483595

[37] Pellegrino M Bellacchio E Dhamo R Frasca F Betterle C Fierabracci A. A novel homozygous mutation of the AIRE gene in an APECED patient from Pakistan: case report and review of the literature. Front Immunol. (2018) 9:1835. 10.3389/fimmu.2018.01835 30150985 PMC6099424

[38] Sanford E Watkins K Nahas S Gottschalk M Coufal NG Farnaes L. Rapid whole-genome sequencing identifies a novel AIRE variant associated with autoimmune polyendocrine syndrome type 1. Cold Spring Harb Mol Case Stud. (2018) 4:a002485. 10.1101/mcs.a002485 29437776 PMC5983174

[39] Fierabracci A Pellegrino M Frasca F Kilic SS Betterle C. APECED in Turkey: a case report and insights on genetic and phenotypic variability. Clin Immunol. (2018) 194:60–6. 10.1016/j.clim.2018.06.012 30018023

[40] Bjorkander JF Brodszki N Carlsson A Fasth A Hansen S Isaksson-Nordmar AN Best possible treatment for all patients with Primary Immune Deficiency (PID) in Sweden regardless of social factors, sex, age or residence. J Allergy Clin Immunol. (2017) 139:AB249 10.1016/j.jaci.2016.12.800

