# Supplementary figures and images for: Primary Immunodeficiencies in Russia: Data From the National Registry
Source: Front Immunol. 2020 Aug 6;11:1491. doi: 10.3389/fimmu.2020.01491 (PMC7424007; doi:10.3389/fimmu.2020.01491)

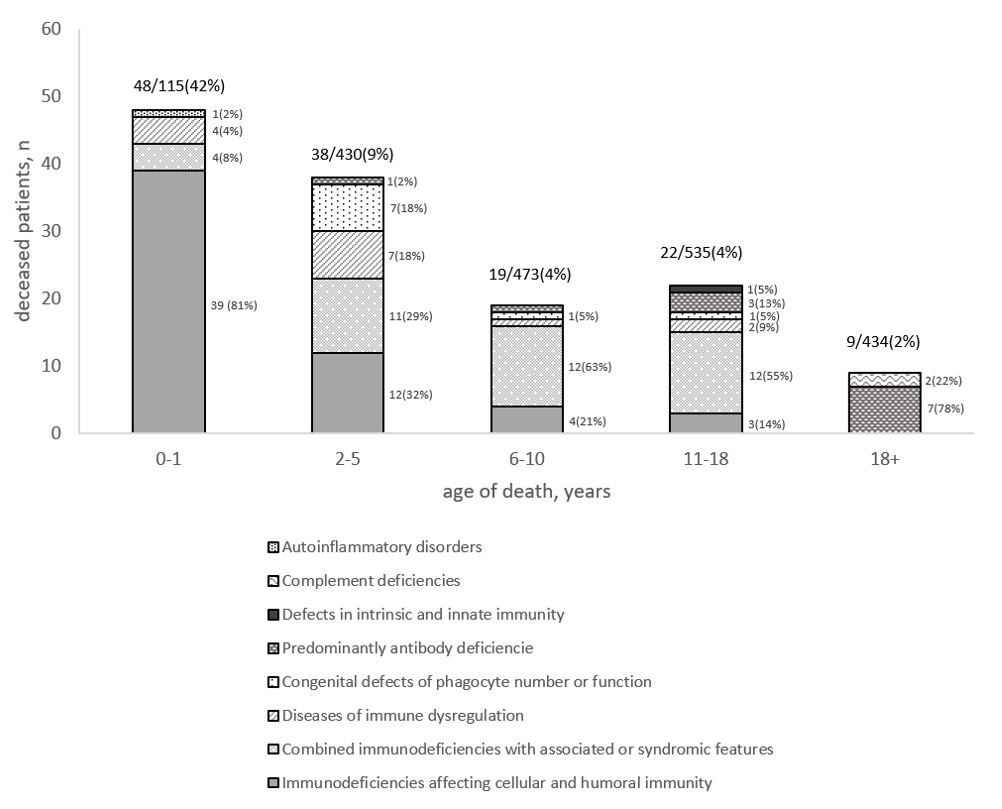

Supplement: Figure S1 — Mortality rate by PID group and age of the deceased patients. Mortality rate in each age group is shown on the top of the stacked bars as a ratio of deceased patients to all patients of the relevant age. Textures represent different PID groups. Numbers next to the plots show the number of patients and the ratio of the deceased patients by PID group to the total number of the deceased patients of the age group. [file Image_1.JPEG]
